# The Many Faces of Immune Thrombocytopenia: Mechanisms, Therapies, and Clinical Challenges in Oncological Patients

**DOI:** 10.3390/jcm13226738

**Published:** 2024-11-08

**Authors:** Marek Kos, Piotr Tomaka, Paulina Mertowska, Sebastian Mertowski, Julia Wojnicka, Anna Błażewicz, Ewelina Grywalska, Krzysztof Bojarski

**Affiliations:** 1 Department of Public Health, Medical University of Lublin, 20-400 Lublin, Poland; 2 Department of Anesthesiology and Intensive Care, SP ZOZ in Łęczna, 21-010 Łęczna, Poland; 3 Department of Experimental Immunology, Medical University of Lublin, 20-093 Lublin, Poland; 4 Department of Pathobiochemistry and Interdisciplinary Applications of Ion Chromatography, Medical University of Lublin, 20-093 Lublin, Poland; 5 General Surgery Department, SP ZOZ in Łęczna, 21-010 Łęczna, Poland

**Keywords:** autoimmune mechanisms, immunosuppressive treatment, ITP, cancer, immune system

## Abstract

The pathogenesis of immune thrombocytopenia (ITP) is complex and involves the dysregulation of immune cells, such as T and B lymphocytes, and several cytokines that promote the production of autoantibodies. In the context of cancer patients, ITP can occur in both primary and secondary forms related to anticancer therapies or the disease itself. Objective: In light of these data, we decided to prepare a literature review that will explain the classification and immunological determinants of the pathogenesis of ITP and present the clinical implications of this condition, especially in patients with cancer. Materials and methods: We reviewed the literature on immunological mechanisms, therapies, and challenges in treating ITP, particularly on cancer patients. Results: The results of the literature review show that ITP in cancer patients can be both primary and secondary, with secondary ITP being more often associated with anticancer therapies such as chemotherapy and immunotherapy. Innovative therapies such as TPO-RA, rituximab, Bruton’s kinase inhibitors, and FcRn receptor inhibitors have shown promising results in treating refractory ITP, especially in patients with chronic disease. Conclusions: ITP is a significant clinical challenge, especially in the context of oncology patients, where both the disease and treatment can worsen thrombocytopenia and increase the risk of bleeding complications. Treatment of oncology patients with ITP requires an individualized approach, and new therapies offer effective tools for managing this condition. Future research into immunological mechanisms may bring further advances in treating ITP and improve outcomes in cancer patients.

## 1. Introduction

Immune thrombocytopenia (ITP) is an acquired autoimmune disorder in which the patient’s body produces autoantibodies directed against its platelets, leading to their destruction and reduced number (thrombocytopenia) [1,2,3,4]. The incidence of ITP varies depending on the geographical region and population, but there is no clear evidence of significant differences between different areas of the world. ITP does not show a preference for specific ethnic or racial groups [5]. Epidemiological data indicate that the annual incidence of ITP in Europe ranges from 1.6 to 3.9 cases per 100,000 people. A wider range is covered by global data, where the incidence ranges from 9.5 to 23.6 cases per 100,000 people per year. In Poland, according to the PLATE study conducted in 2011, the annual incidence of ITP was 3.5 per 100,000 people. ITP can occur in people of all ages and genders, but incidence patterns indicate a higher incidence in women aged 30–60 years. In children, the disease occurs at a frequency of 1.9–6.4 per 100,000 children per year, with the highest incidence in children under ten years of age. In men, an increased risk of disease onset is observed in childhood and over 60 [5,6,7,8,9,10,11].

**Figure 1:**
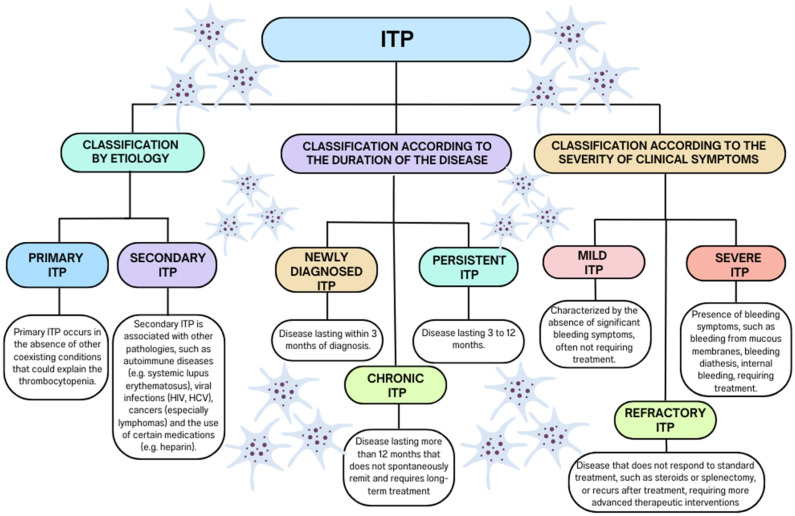
Classification of ITP according to symptoms, type, and duration of the disease (based on [15,16,17,18,19,20,21]).

In ITP, there is an imbalance between platelet destruction and production [12,13,14]. The main mechanism leading to thrombocytopenia is the coating of platelets by autoantibodies, which facilitates their recognition by Fcγ receptors on the surface of macrophages. This process leads to platelet phagocytosis and their destruction, mainly in the spleen and liver. Additionally, antibodies can inhibit the function of megakaryocytes in the bone marrow, which leads to a decrease in platelet production. In recent years, studies have also shown the involvement of T cells (both cytotoxic and regulatory) in the pathogenesis of ITP. In particular, defects in the function of regulatory T cells (Treg) contribute to the maintenance of autoimmune reactions [15,16,17]. This complex autoimmune disorder arises from multifactorial pathogenesis and diverse pathophysiological mechanisms, leading to variable clinical presentations. Accurate classification, based on disease duration, symptom severity, and comorbidities (Figure 1), is crucial for understanding its progression and optimizing treatment strategies. This disorder presents significant clinical challenges, such as increased bleeding risk, treatment resistance, and a profound impact on the patient’s quality of life. To address these issues, a personalized approach and long-term management are essential for reducing complications and improving therapeutic outcomes [18,19,20,21].

Patients most at risk of serious complications associated with ITP are primarily those with very low platelet counts (<20,000/µL), which increases the risk of life-threatening internal bleeding, but also elderly patients with comorbidities such as hypertension or heart disease and oncology patients. Oncological patients are a group particularly predisposed to the development of severe complications associated with ITP. Both the presence of cancer and anticancer treatment, including chemotherapy, significantly increase the risk of thrombocytopenia, which in turn increases the likelihood of bleeding. Additionally, surgical procedures, such as resection of neoplastic tumors, pose a particular challenge for these patients due to the accompanying thrombocytopenia, which significantly increases the risk of bleeding complications both during and after surgery [18,22,23]. This literature review aims to provide a detailed discussion of the immunological determinants of ITP pathogenesis and the clinical implications of this condition, with particular emphasis on cancer patients. This group is particularly susceptible to the development of serious complications associated with ITP, resulting from both the presence of cancer and anticancer treatment. The review aims to provide a comprehensive understanding of the factors increasing the risk of thrombocytopenia in cancer patients and the impact of thrombocytopenia on the course of therapy and surgical procedures, which may contribute to better management of bleeding risk in this patient population.

## 2. Pathogenesis of ITP

ITP is a heterogeneous autoimmune disorder that can occur in primary or secondary forms. The distinction between these forms is crucial for developing an effective therapeutic strategy. Primary ITP is characterized by thrombocytopenia resulting from unknown causes, without comorbidities. In this form, thrombocytopenia results from autoimmune destruction of platelets by autoantibodies. Secondary ITP, on the other hand, develops as a result of clearly defined factors, such as autoimmune diseases (e.g., systemic lupus erythematosus), viral infections (e.g., human immunodeficiency virus (HIV) and hepatitis C virus (HCV)), hematological malignancies (e.g., lymphomas and leukemias), or drugs (e.g., heparin). A detailed comparison of primary and secondary ITP is provided in Table 1.

**Figure 2:**
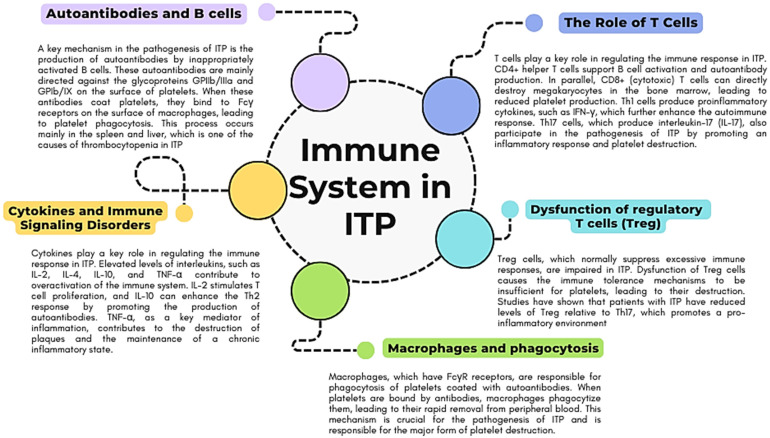
The most important immune system dysfunctions contributing to the development of ITP (based on [14,24,25,26,27,28,29,30]).

A key element of the pathogenesis of ITP, both in its primary and secondary forms, is the dysregulation of the immune system, which leads to the production of autoantibodies against platelet surface antigens and, consequently, to their destruction [24]. This process involves abnormalities in the activation and regulation of B and T cells, cytokines, and macrophages, which, in combination with other immunological mechanisms, leads to a shortened platelet lifespan and increased thrombocytopenia (Figure 2).

### 2.1. The Role of B and T Lymphocytes in the Pathogenesis of ITP

In hematological malignancies such as non-Hodgkin lymphomas (NHL) and chronic lymphocytic leukemia (CLL), immune disorders are particularly severe, leading to the development of secondary ITP. In these cases, B lymphocytes have excessive activation, which produces autoantibodies directed against platelet surface antigens, such as glycoproteins GPIIb/IIIa. These autoantibodies coat platelets, which leads to their recognition by Fcγ receptors on macrophages and initiation of their phagocytosis, mainly in the spleen. This process causes rapid platelet destruction, leading to thrombocytopenia. At the same time, there is an imbalance between the B and T lymphocyte populations in CLL [37,38]. A decrease in regulatory T lymphocytes (Treg), which normally inhibit excessive immune activation, promotes autoimmunity, and increases platelet destruction. Dysfunction of these regulatory T cells combined with excessive B cell activity leads to a persistent, pathological immune response resistant to standard treatment methods [39,40,41,42]. An additional factor exacerbating the course of secondary ITP in hematological malignancies is changes in the bone marrow microenvironment. Cancers such as CLL and lymphomas can cause neoplastic infiltration in the bone marrow, disrupting normal hematopoiesis, including the maturation of megakaryocytes—cells responsible for platelet production. Inhibition of new platelet production by megakaryocytes and their excessive destruction due to autoimmune processes deepens thrombocytopenia, making treatment particularly difficult and requiring a multifaceted approach [43,44,45].

Recent studies have shown that autoreactive T cells play a key role in initiating and perpetuating the autoimmune response by helping B cells produce autoantibodies. CD4+ T cells and CD8+ T cells (cytotoxic T cells) that can destroy megakaryocytes, macrophages, dendritic cells, and dysfunctional regulatory T cells (Tregs), which are normally responsible for suppressing the immune response and maintaining peripheral tolerance, have been implicated in the pathogenesis of ITP, suggesting impaired function of Tregs in patients with this disease, as well as regulatory B cells (Bregs) [14,25,26,27,28,29,30].

In addition, an imbalance in Th17, Th1, Th2, and Th3 may contribute to an increased pro-platelet environment and trigger the process of platelet destruction. Studies show that in ITP, Treg cells are reduced compared to Th17, which promotes inflammation [4,46,47,48,49,50,51]. Aberrant cytokine signaling, particularly elevated levels of interleukin-2 (IL-2), interleukin-4 (IL-4), interleukin-10 (IL-10), and tumor necrosis factor-alpha (TNF-α), further perpetuates immune activation and platelet destruction in ITP [15,27,52,53]. Studies on immunoregulatory gene polymorphisms in patients with ITP indicate significant differences in the susceptibility to developing the disease depending on the presence of specific mutations. By analyzing cytokines and their receptors, several genes, such as *TNFA*, *IL-1B*, *IL-10*, and *IL-17F*, have been identified as key in the pathogenesis of ITP. Studies conducted in different ethnic groups, including Japanese, Chinese, Turkish, and Egyptian, have shown that polymorphisms in these genes may influence the development and severity of ITP. For example, in the Chinese population, polymorphisms in the *IL-23R* and *IL-18* genes were associated with different types of ITP, both acute and chronic. In the Japanese population, polymorphisms in the *TNFA* and *IL-17F* genes were associated with chronic ITP [53].

The role of regulatory T cells in ITP has also been investigated regarding genes such as *CTLA4* and *PDCD1*, which control T cell activation. In Asian and Caucasian populations, different variants of these genes have been identified that are associated with the regulation of the immune response in ITP. For example, polymorphisms in the *CTLA4* gene are associated with impaired regulation of T lymphocytes, which may lead to excessive autoimmune reactions and increased platelet destruction. In cancer patients, especially those treated with immunotherapies such as antibodies against CTLA-4 (e.g., ipilimumab), increased activation of T lymphocytes is observed. Similar to ITP, inhibition of *CTLA4* function leads to reduced immune tolerance, which may promote the development of autoimmune reactions against one’s cells, including platelets. Therefore, cancer patients undergoing such therapies are at increased risk of secondary autoimmune complications, including ITP. Similar mechanisms apply to the *PDCD1* gene, which encodes the PD-1 receptor responsible for inhibiting excessive activation of T cells. Blocking this pathway with checkpoint inhibitors in cancer therapy may lead to immune dysregulation, contributing to the autoimmune destruction of platelets, especially in the context of secondary ITP [54,55].

### 2.2. The Role of Genetic Polymorphisms and Epigenetic Modifications in the Pathogenesis of ITP

Significant polymorphisms have also been identified in genes encoding FcγR receptors, such as *FCGR2A* and *FCGR3A*, which regulate autoantibodies’ binding to platelets, affecting their phagocytosis by macrophages. These polymorphisms were particularly significant in Caucasian, Asian, and African populations, where they were shown to be associated with the severity and chronicity of ITP [53].

Emerging evidence suggests that epigenetic modifications, such as DNA methylation and histone acetylation, play a key role in the pathogenesis of ITP by regulating gene expression patterns and immune responses. Abnormal DNA methylation patterns have been observed in genes involved in immune regulation and platelet function in patients with ITP, suggesting a potential role of epigenetic dysregulation in shaping the autoimmune and thrombocytopenic disease phenotype [56,57]. In the context of cancer patients, these epigenetic mechanisms are of particular importance, as cancers are often characterized by aberrant DNA methylation patterns. In patients with ITP, abnormal methylation patterns of genes involved in immune regulation and platelet function have been observed, suggesting that epigenetic dysregulation may exacerbate symptoms of both cancer and autoimmune disease. Hypermethylation of tumor suppressor gene promoters in cancer cells and hypomethylation of proliferative genes may lead to increased tumor activity while at the same time deepening the immune dysfunctions characteristic of ITP [58,59]. Additionally, epigenetic modifications, such as histone acetylation, affect the expression of genes responsible for the immune response, which in cancer patients may lead to chronic activation or suppression of the immune system. This, in turn, may enhance autoimmune platelet destruction, worsening the course of ITP [60,61,62]. Chemotherapy, widely used in cancer treatment, can also affect the epigenome, causing changes in DNA methylation and histone acetylation, which affect hematological functions, including platelet production. In patients with ITP, these changes can further exacerbate disease symptoms, leading to difficulties in controlling thrombocytopenia. Immunotherapy, also increasingly used in cancer treatment, can modulate epigenetic patterns, potentially increasing autoimmune reactions against platelets. For this reason, cancer patients with ITP require special attention because therapies that affect the epigenome can both support cancer control and worsen ITP symptoms [63,64,65,66].

Moreover, some studies indicate that aberrant expressions of miRNAs, especially miR-155, miR-146, miR-142, and miR-181, also play an important role in the pathophysiology of ITP [67]. Moreover, they also play a significant role in the development and progression of cancer. miR-155 is classified as an “oncomiR” because it is overexpressed in various types of cancers, such as lung cancer, lymphoma, and breast cancer. MiR-155 promotes cancer cell proliferation, supports tumor survival, and contributes to metastasis by regulating key pathways related to immune response, inflammation, and cell migration. High levels of miR-155 are also associated with a poorer prognosis in cancer patients [68,69]. miR-146 and miR-146a play a more complex role by regulating inflammatory responses and immune mechanisms. In the context of cancer, they can act as both tumor suppressors and tumor promoters, depending on the specific conditions. In hematological malignancies, such as leukemia, miR-146a regulates inflammatory pathways, which contribute to disease progression [70,71]. miR-181 is associated with promoting cell cycle progression in cancer cells, particularly in choroidal melanoma, whereby inhibiting tumor suppressor genes, miR-181 accelerates cancer cell proliferation, leading to more aggressive forms of cancer [72]. In ITP, the same miRNAs, particularly miR-155 and miR-146, affect immune dysfunction. Overexpression of miR-155 in ITP leads to increased immune system activity, which promotes the production of autoantibodies directed against platelets. In turn, miR-146a is involved in the negative regulation of immune responses, and its dysregulation in ITP may disturb the balance between pro-inflammatory and anti-inflammatory signals, which further enhances platelet destruction.

### 2.3. The Role of Viral and Bacterial Infections in the Pathogenesis of ITP

Other factors that may also affect megakaryocyte function include, for example, inducing immune responses leading to platelet destruction and disruption of megakaryocyte production. These mechanisms may include direct effects of viruses on megakaryocytes, such as altering their gene expression or inducing apoptosis, and indirect effects mediated by the immune system [73,74]. Among the different types of infections, viral infections stand out as the most common causative agent of this disorder. Viruses can stimulate the immune system to produce antibodies, mistakenly attacking platelets and destroying them. In cancer patients, immune dysregulation caused by viral infections such as HIV, HCV, EBV (Epstein–Barr virus), CMV (cytomegalovirus), or SARS-CoV-2 (severe acute respiratory syndrome coronavirus 2) may play a significant role in the development and progression of ITP. Oncological therapy, especially chemotherapy and immunotherapy, leads to immunosuppression, which increases susceptibility to viral infections. These viruses can induce significant changes in the functioning of T and B lymphocytes, resulting in an abnormal immune response, including the production of autoantibodies directed against platelets [47,75,76].

In the case of HIV infection, the virus directly infects CD4+ T cells, which are crucial for maintaining immune homeostasis. In HIV patients, there is a profound impairment of regulatory T cell (Treg) function, leading to the overproduction of autoantibodies against platelets by activated B cells. HIV-infected cancer patients are at increased risk of developing ITP due to the cumulative effect of immunosuppression resulting from viral infection and cancer therapy. This process may be further exacerbated by viral replication in the setting of immunosuppression, which causes increased production of autoantibodies and increases platelet destruction [77,78].

The EBV, an oncogenic virus, plays a special role in the pathogenesis of various hematological diseases and neoplasms, including lymphomas, nasopharyngeal carcinoma, and malignant transformation of B lymphocytes. Its oncogenic properties result from its ability to infect B lymphocytes, where it can cause polyclonal activation and uncontrolled proliferation of these cells. As a result, EBV is a risk factor for the development of lymphomas in immunocompromised patients, such as transplant patients or oncology patients. In the context of ITP, EBV infection can lead to polyclonal activation of B lymphocytes, resulting in the production of autoantibodies against platelet surface antigens such as GPIIb/IIIa. These autoantibodies can induce their opsonization and phagocytosis by macrophages, leading to the destruction of platelets in the spleen. In cancer patients, especially those struggling with hematological malignancies, EBV infection can also lead to the development of so-called lymphoproliferative syndromes associated with this virus. In such cases, viral replication and its oncogenic effects not only contribute to the development of lymphomas but can also exacerbate existing immune dysfunctions, including the development of ITP. Since EBV can remain latent in B lymphocytes, cancer patients are more susceptible to its reactivation as a result of weakened immunity, which additionally increases the risk of developing ITP. In patients with EBV-related cancers, interactions between the oncogenic properties of the virus and immune dysregulation can significantly complicate the treatment and control of thrombocytopenia. In such cases, a multifaceted therapeutic strategy is necessary, including control of viral infections and careful monitoring of platelet function [79,80,81].

COVID-19 infection can lead to the development of ITP due to excessive activation of the immune system. In severe cases of COVID-19, a so-called cytokine storm is observed, during which the body produces excessive amounts of pro-inflammatory cytokines such as IL-6, IL-1β, TNF-α, and interferon-gamma (IFN-γ). Excessive activation of these inflammatory pathways leads to immune dysregulation, including inappropriate activation of T and B lymphocytes. B lymphocytes can then produce autoantibodies directed against platelets, which cause their destruction by macrophages in the spleen. In addition, evidence suggests that SARS-CoV-2 can directly affect megakaryocytes, or platelet precursor cells, in the bone marrow, disrupting their function and platelet production and further deepening thrombocytopenia. In severe cases of COVID-19, thromboembolic complications are also frequently observed, which can lead to platelet consumption during clot formation while predisposing patients to bleeding due to thrombocytopenia [82,83,84].

In oncology patients who are undergoing intensive immunosuppression associated with chemotherapy, radiotherapy, or immunotherapy, the risk of ITP due to SARS-CoV-2 infection is significantly increased. Oncotherapy significantly weakens the immune system, making these patients more susceptible to viral infections, including SARS-CoV-2 infection. Additionally, immunosuppression can intensify the inflammatory response and promote the production of autoantibodies against platelets, leading to their increased destruction. SARS-CoV-2 infection in oncology patients not only increases the risk of severe COVID-19 but can also trigger an excessive immune response, leading to a cytokine storm, which in turn promotes the development of ITP. In these patients, both viral infection and anticancer therapies may cause platelet destruction and disrupt their production, leading to serious clinical consequences such as severe thrombocytopenia and associated risk of bleeding [85,86].

Although viral infections are the most common cause of ITP, bacterial infections may also contribute to its development. One of the best-studied examples is chronic infection with the bacterium *Helicobacter pylori*, known mainly for causing gastritis and peptic ulcers. In patients with ITP, coinfection with *H. pylori* is often observed, and its eradication in many cases leads to an improvement in platelet counts. The mechanism underlying this phenomenon is probably related to molecular mimicry—*H. pylori* antigens can mimic platelet structures, leading to an erroneous immune response and production of autoantibodies directed against platelets. Another possible mechanism is general immune dysregulation, in which chronic *H. pylori* infection induces abnormal activation of immune cells, which in turn leads to platelet destruction [87,88,89]. In the context of cancer patients, infections caused by *Streptococcus pneumoniae* may play a particularly important role in the development of ITP. Cancer patients are often more susceptible to bacterial infections, including pneumococcal infections, due to a weakened immune system caused by both the cancer itself and the therapy used, such as chemotherapy, radiotherapy, or immunosuppressive treatment. As a result of the weakened immune system, even typical bacteria such as *S. pneumoniae* can cause severe infections, which additionally complicate the course of the cancer disease [90]. Bacterial infections such as pneumonia, sepsis, or meningitis can induce an acute, excessive immune response. In such a state, the body mobilizes a large number of immune cells, including B and T lymphocytes, which can lead to the production of autoantibodies directed against platelets. In cancer patients, due to their weakened immune system, this immune response may be more intense, and the mechanisms of immune response control are often impaired. This means that these patients are more susceptible to excessive autoimmune reactions, including the development of ITP, which results in the misidentification of platelets as pathogens [91].

The mechanism of ITP development in the course of *S. pneumoniae* infection in cancer patients may resemble the mechanism observed in viral infections. In response to bacterial infection, the immune system is overactivated, leading to the production of autoantibodies against platelet surface antigens, such as glycoproteins GPIIb/IIIa. These autoantibodies bind to platelets, which are then removed by macrophages in the spleen or other parts of the reticuloendothelial system. This process leads to a decrease in platelet counts, which can cause symptoms of thrombocytopenia, such as the tendency to bruise, nosebleeds, or internal bleeding [49]. For immunocompromised cancer patients, pneumococcal infections pose a particular threat because they not only can cause ITP but also exacerbate existing complications related to thrombocytopenia. In such cases, rapid identification of the infection, aggressive antibiotic therapy, and appropriate control of immunosuppression are crucial to reduce the risk of further platelet destruction. ITP therapy in cancer patients often also requires the use of immunoglobulins, corticosteroids, or immunosuppressive drugs, which further complicates treatment, especially in the context of concomitant bacterial infections [49].

### 2.4. Nutrient Deficiencies in the Pathogenesis of ITP

In the context of ITP, vitamin deficiencies, especially vitamin B12 and folic acid, can significantly affect platelet production and worsen the symptoms of thrombocytopenia. Vitamin B12 and folic acid are essential for DNA synthesis, and their deficiencies lead to ineffective hematopoiesis, that is, disorders in the production of all cell lines in the bone marrow, including megakaryocytes, which are the precursors of platelets. In the case of a deficiency of these vitamins, platelet production is reduced, which can lead to increased symptoms of ITP, such as the increased tendency to bleed, petechiae, bruises, and internal hemorrhages [75,76,92,93,94,95,96]. Vitamin B12 plays a key role in nucleotide synthesis and homocysteine metabolism and also affects the proper maturation of megakaryocytes. Deficiency of this vitamin leads to impaired maturation of megakaryocytes, which results in reduced platelet production. In patients with ITP, where autoimmune mechanisms lead to platelet destruction, additional reduction in their production due to vitamin B12 deficiency may exacerbate thrombocytopenia. Megaloblastic anemia resulting from vitamin B12 deficiency not only causes disorders in red blood cell production but also reduces the number of platelets, which is particularly problematic in patients with ITP [92,93].

Folic acid, a key component necessary for the synthesis of purines and pyrimidines and DNA methylation processes, also has a significant impact on platelet production. Its deficiency may lead to abnormal maturation of megakaryocytes and a decrease in platelet count, which intensifies the symptoms of ITP. In the bone marrow, where intensive production of blood cells occurs, a lack of folic acid may lead to disorders of hematopoiesis, which, in combination with autoimmune mechanisms in ITP, may lead to more severe forms of thrombocytopenia [97,98].

In the context of cancer patients with ITP, vitamin B12 and folate deficiencies are of particular importance. Anticancer therapy, including chemotherapy and radiotherapy, often leads to impaired absorption of these vitamins, which can exacerbate nutritional deficiencies and deepen thrombocytopenia. Damage to the gastrointestinal epithelium resulting from chemotherapy can lead to reduced absorption of vitamin B12 and folate, which additionally burdens the hematopoietic system. Cancer patients may suffer from chronic inflammation, weight loss, and cachexia, which increases the risk of deficiencies of these key vitamins. As a result, hematopoiesis is impaired, which results in a decrease in platelet count and exacerbation of ITP symptoms [99,100,101].

In addition, in patients with gastrointestinal cancers, such as stomach, intestinal, or pancreatic cancer, impaired absorption of vitamin B12 and folate may be even more severe. Reduced availability of these vitamins may lead to even greater hematopoietic deficits, which may further complicate the treatment of patients with ITP. These patients are at increased risk of severe complications associated with thrombocytopenia, such as internal hemorrhage, internal bleeding, and skin bruising [102,103,104,105,106,107,108].

### 2.5. Drugs Involved in the Development of ITP

Medications can be a significant risk factor for developing secondary ITP. Although not every patient taking medication develops ITP, some medications are associated with an increased risk of developing the condition [8,24]. One of the most well-known examples is heparin. This popular anticoagulant works by increasing the activity of antithrombin III, a natural coagulation inhibitor that inhibits coagulation factors such as thrombin (factor IIa) and factor Xa. Sometimes, heparin can cause heparin-induced thrombocytopenia (HIT), which is based on forming immune complexes with platelet factor 4 (PF4). The binding of heparin to PF4 leads to changes in the structure of this protein, which causes the exposure of new epitopes that the immune system recognizes as foreign. IgG antibodies are produced that bind to the heparin-PF4 complex, activating platelets, which can lead to their destruction and the risk of thrombosis. In rare cases, HIT can progress to ITP due to platelet consumption and destruction [109,110,111,112].

In addition to heparin, other drugs can also cause secondary ITP through different mechanisms. Antibacterials such as sulfonamides, beta-lactam antibiotics, and vancomycin, an antibiotic used to treat infections caused by Gram-positive bacteria, including MRSA (methicillin-resistant *Staphylococcus aureus*), have been associated with thrombocytopenia [113,114,115,116,117,118]. Rifampicin, used to treat tuberculosis, and trimethoprim-sulfamethoxazole, widely used to treat urinary tract and lung infections, can also contribute to thrombocytopenia. In these cases, the mechanism may involve the formation of antigen–antibody complexes or direct effects on immune cells [119,120]. Anticonvulsants such as valproic acid and carbamazepine and some nonsteroidal anti-inflammatory drugs (NSAIDs), including acetaminophen and diclofenac, have also been associated with immune thrombocytopenia. However, the exact mechanism is not fully understood [121,122,123,124,125,126,127].

Some chemotherapy agents can cause ITP through various mechanisms, including bone marrow damage, immune dysfunction, and the formation of antigen–antibody complexes. Drugs that are often associated with thrombocytopenia include gemcitabine and mitomycin-C. These drugs can damage the vascular endothelium, resulting in hemolytic uremic syndrome (HUS), including thrombocytopenia. In addition, vancomycin, used to treat bacterial infections, and rifampicin, used to treat tuberculosis, can also cause thrombocytopenia through bone marrow toxicity or autoimmune mechanisms. The mechanism of action of these drugs often involves the activation of the immune system, leading to the production of antibodies against platelets or their direct destruction. In the case of chemotherapy-induced thrombocytopenia, up to 60% of cancer patients may experience a decrease in platelet counts, which may require dose adjustments or delays in subsequent treatment cycles [128,129,130,131,132].

## 3. ITP in Cancer

**Figure 3:**
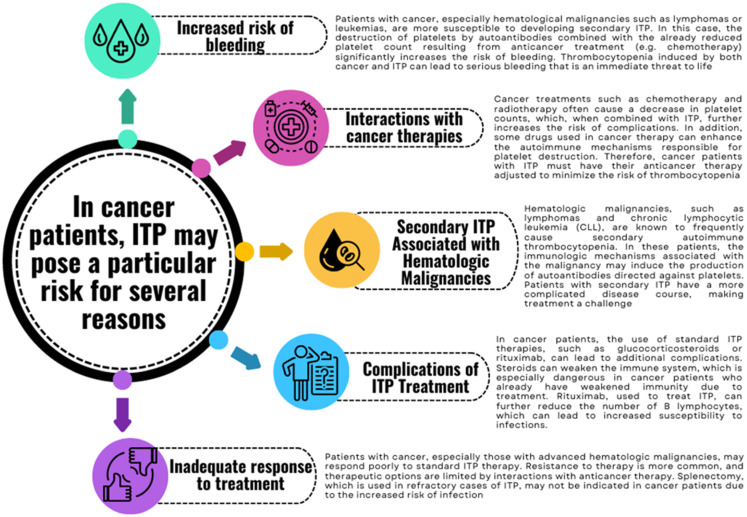
Risks of developing ITP in cancer patients (based on [139,140]).

ITP poses a significant risk to certain patient populations, particularly those with severe disease or comorbid conditions (Figure 3). Cancer patients, in particular, warrant special attention. In this group, ITP can manifest in both primary and secondary forms, with secondary ITP being more prevalent [54]. Primary ITP in cancer patients is less common, and its mechanism is not directly associated with the tumor or its treatment. Instead, it may result from an idiopathic autoimmune reaction that leads to platelet destruction by autoantibodies, without a clear link to the malignancy. Nevertheless, secondary forms of thrombocytopenia are more frequent in cancer patients, often due to the presence of comorbidities and the effects of anticancer therapies. Certain solid tumors, particularly gastrointestinal cancers [133], cancers of the reproductive system, and kidney cancers [134], as well as malignancies with bone marrow metastases, such as breast and lung cancer, are strongly associated with the development of secondary ITP. In these cases, neoplastic infiltration of the bone marrow can result in pancytopenia, including thrombocytopenia, by directly damaging hematopoietic precursor cells. Furthermore, immune mechanisms, such as autoantibody production, can aggravate thrombocytopenia, leading to secondary ITP [135,136,137]. Immunosuppression caused by anticancer therapies, such as chemotherapy and radiotherapy, further compromises the immune system, facilitating the development of autoimmune reactions that target platelets [128,138]. Some chemotherapeutic agents cause ITP through bone marrow damage, immune dysregulation, or antigen–antibody complexes. Gemcitabine, mitomycin-C, vancomycin, and rifampicin can trigger thrombocytopenia via vascular damage or immune reactions. This often leads to platelet destruction and affects up to 60% of cancer patients, requiring treatment adjustments [128,129,130,131,132].

### 3.1. Primary ITP in Oncology Patients

An example is the case report of a patient with breast cancer who developed primary ITP. In this situation, the patient underwent standard anticancer treatment, but the accompanying thrombocytopenia was of autoimmune origin, not directly related to the therapy or the presence of metastases. Treatment of ITP included standard immunosuppressive therapies, such as the administration of corticosteroids, which led to the stabilization of the platelet count [139].

Studies conducted in the Swedish population show that patients with primary ITP have a higher risk of developing various types of cancer compared to the general population without ITP. The overall risk of cancer was higher in patients with ITP (cancer incidence rate ratio in patients with ITP compared to those without ITP, i.e., IRR = 1.45), with the risk being higher in men (IRR = 1.60) than in women (IRR = 1.33). One group of cancers in which a significantly increased risk was observed is gastrointestinal cancer. Patients with ITP have an increased risk of gastrointestinal cancer, particularly liver cancer, where the IRR was 5.88, and in men, the risk is as high as 6.56. An increased risk is also observed in the case of colorectal cancer (IRR = 1.39), especially in men, where the risk ratio is 1.67. Another area in which the risk of disease is significantly higher is hematological cancer. The risk of developing myeloid leukemia in patients with ITP is eight times higher (IRR = 8.39), with women being even more at risk for this type of cancer (IRR = 14.31). Similarly, an increased risk is observed in the case of lymphomas, with an overall IRR of 4.63. In the case of skin cancers, an increased risk was also noted in patients with ITP (IRR = 1.37), with higher risk ratios in men (IRR = 1.52). On the other hand, women with ITP have a reduced risk of breast cancer (IRR = 0.80). The risk of developing some organ cancers was increased only in the first year after ITP diagnosis. This concerned colon cancer (hazard ratios of HR 3.45), rectal cancer (HR 3.78), ovarian cancer (HR 3.60), and brain cancer (HR 5.36). On the other hand, the risk of liver cancer increased only in 2–9 years after diagnosis (HR 5.03). An increased risk of hematologic malignancies, including lymphomas, was evident in all periods. In men, the risk of myeloid leukemia persisted for up to 9 years, and in women, it significantly increased between 2 and 20 years after diagnosis. The increased risk of skin cancer did not occur until 10–20 years after the diagnosis of ITP. The results remained unchanged after excluding patients with splenectomy. These data suggest a significant association between ITP and an increased risk of cancer, particularly in the context of hematologic and gastrointestinal malignancies. Patients with ITP, both men and women, require intensive monitoring for early signs of cancer, allowing for earlier diagnosis and intervention [140].

### 3.2. Secondary ITP in Oncology Patients

Cancer patients, especially those with hematological malignancies such as lymphoma, leukemia, and multiple myeloma, are at increased risk of developing secondary ITP. This is due to the complex dysregulation of the immune system that accompanies the cancer and the treatments that patients receive. In hematological malignancies, cancer cells can affect the immune system, producing autoantibodies that attack platelets. This process can be more severe in patients with lymphomas and leukemias, in which the immune system is already weakened or overactive [39,40,41,42,141,142,143].

The mechanisms of this phenomenon are twofold. First, hematological malignancies themselves can trigger autoimmune reactions. The changes in the B and T lymphocyte populations characteristic of these tumors lead to an impairment of the normal immune response and the production of autoantibodies against platelet antigens. Increased B-cell activity in leukemias and dysregulation of T-cells can enhance platelet destruction via autoimmune mechanisms, resulting in thrombocytopenia and increased risk of bleeding [35,144,145]. Second, some cancer therapies increase the risk of developing ITP. Immunotherapy, particularly immune checkpoint inhibitors (ICIs) such as pembrolizumab and nivolumab, used to treat melanoma, lung cancer, and other cancers, has been associated with cases of severe thrombocytopenia, which can lead to ITP. Although checkpoint inhibitors enhance the body’s immune response to cancer cells, they can also trigger an excessive autoimmune response that damages platelets. Severe thrombocytopenia (e.g., grade 3 or higher thrombocytopenia) has been reported in patients treated with ICIs, which further worsens the symptoms of ITP and can lead to serious bleeding. In addition to immunotherapy, other anticancer therapies, such as chemotherapy, also increase the risk of developing ITP [146,147,148]. Chemotherapy can lead to direct damage to the bone marrow, impairing platelet production, which further increases the risk of thrombocytopenia. Drugs such as gemcitabine and mitomycin C are known to cause severe thrombocytopenia through toxic and autoimmune mechanisms, which, combined with the weakened immune system of cancer patients, can lead to an exacerbation of ITP [128,129,130,131,132,133].

**Figure 4:**
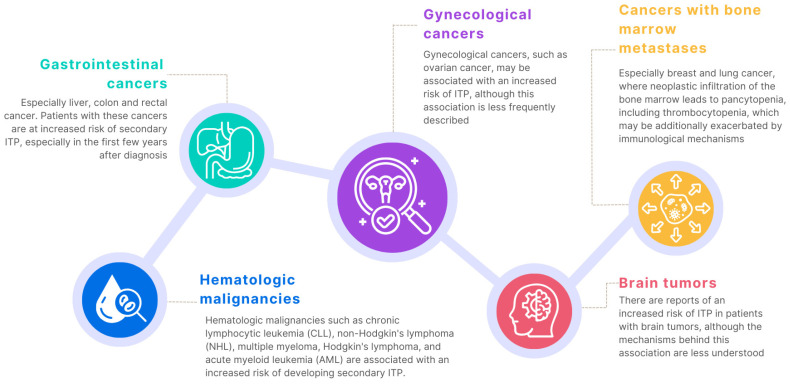
Types of cancers at risk of developing ITP (based on [128,129,130,131,132,133,149,150]).

A similar situation applies to other types of cancer [133,134,135,136,137]. Analysis of literature data has shown that patients with gastrointestinal cancers, such as stomach, colon, or liver cancer, are also at risk of developing ITP (Figure 4).

This risk is a result of both the nature of gastrointestinal cancers and the treatment used to treat these diseases, including chemotherapy and immunotherapy, as is the case with ICI therapy. In the case of the first two types of cancers (stomach and colon cancer), which are often diagnosed at advanced stages, the risk of complications, including thrombocytopenia, is increased. Patients with stomach cancer may be at risk of developing ITP, especially in cases of advanced disease or after surgical treatment. Several cases have been described in which ITP occurred in association with stomach cancer, especially in patients with advanced cancer and in those who underwent gastrectomy combined with splenectomy [133,149,150]. In one case of a patient with gastric cancer and ITP, radical gastrectomy and splenectomy improved platelet counts and stabilized the patient’s condition, although it was a high-risk procedure, especially because of the risk of major bleeding associated with ITP [151]. Gastric cancer, especially in the case of liver metastases, can lead to coagulation disorders, increasing the tendency to bleed and autoimmune destruction of platelets. In patients with colon cancer, the risk of ITP is higher in the first years after diagnosis, especially in the case of liver dissemination [152,153,154,155]. Although in the case of colon cancer, ITP is a rarer complication but can still occur in the context of cancer or anticancer therapy, it has been shown that patients treated with fluorouracil (5-FU) or oxaliplatin are at greater risk of thrombocytopenia, which may promote the development of ITP. Complications related to thrombocytopenia in these patients can lead to serious bleeding, especially during cancer surgery [156,157]. Gastrectomy, used to treat gastric cancer, may contribute to the development of ITP through several complex immunological and physiological mechanisms. This surgery causes systemic stress that activates the immune system, leading to increased production of proinflammatory cytokines such as IL-6, TNF-α, and IL-1. These mediators can stimulate antigen-presenting cells, which in turn lead to the production of autoantibodies against platelets, key in the pathophysiology of ITP. As a result, the surgery can initiate an autoimmune response leading to platelet destruction. Additionally, gastrectomy carries the risk of postoperative bleeding, which not only reduces the platelet count but may also require blood transfusion. Transfusions, especially repeated ones, can lead to alloimmunization, i.e., the production of antibodies against the antigens of the transfused platelets, which further worsens thrombocytopenia. Therefore, bleeding and its associated treatment may indirectly promote the development of ITP. In addition, after gastrectomy, the gastrointestinal microenvironment is disrupted, which can affect hematopoietic metabolism, further complicating the situation. Stomach surgery can also affect the immunological-surgical plexus, disrupting the body’s immune tolerance. Surgical stress can lead to the production of autoantibodies against platelet surface glycoproteins, which promotes their destruction by the spleen. In cancer patients, who often have a weakened immune system, these mechanisms are more pronounced. Additionally, the removal of the tumor itself can release internal proteins that become new autoantigens, stimulating the immune system to destroy platelets. Stomach cancer, especially in advanced stages, can also contribute to the development of ITP through chronic inflammation, which predisposes to the development of this condition. Surgery can act as a trigger to unmask existing autoimmune mechanisms, increasing platelet destruction by the spleen and other elements of the reticuloendothelial system [133,149,150,151,152,153,154,155,156,157].

Liver cancer patients are particularly susceptible to ITP because liver function has a key impact on the clotting process and platelet production. The liver plays an important role in synthesizing many clotting factors, as well as proteins regulating hemostasis, such as thrombopoietin, a hormone responsible for stimulating platelet production in the bone marrow. In the course of liver cancer, both the case of primary tumors such as hepatocellular carcinoma (HCC) and metastases from other organs such as the stomach or large intestine, liver function is impaired, which can lead to thrombocytopenia and thus increase the risk of bleeding. Chemotherapy and targeted therapy used to treat liver cancer and gastrointestinal cancers can lead to toxic damage to the bone marrow, where platelets are produced. For example, drugs such as bevacizumab, an angiogenesis inhibitor used in cancer treatment, can lead to serious complications, such as bleeding, due to a decrease in platelet count and damage to blood vessels. These drugs can worsen thrombocytopenia, which leads to even more serious complications in patients with concomitant ITP. Bone marrow damage from chemotherapy reduces megakaryocyte production, which further worsens thrombocytopenia [158,159,160,161,162].

Patients with gynecological malignancies, such as ovarian, cervical, or endometrial cancer, may also be at risk for developing ITP. Although ITP is more commonly associated with hematological malignancies, there are cases of it occurring in the context of solid malignancies, including gynecological ones. The mechanism is not fully understood, but it has been suggested that cancer may lead to immune dysregulation, which promotes autoimmune destruction of platelets. In the case of ovarian cancer, ITP may be associated with both cancer and anticancer therapy [160,162,163,164]. Cancer may disrupt the immune balance, leading to inappropriate activation of B and T cells and the production of autoantibodies. Ovarian cancer, which often leads to metastasis and advanced inflammation, may additionally increase autoimmune reactions that lead to platelet destruction. One mechanism that may be associated with the development of ITP is excessive activation of the immune system in response to cancer, which may lead to auto-aggression directed against one’s cells, including platelets [165,166,167]. Treatment for ovarian cancer, especially advanced cancer, often requires intensive chemotherapy. Drugs such as paclitaxel, cisplatin, and carboplatin, commonly used to treat ovarian cancer, can lead to bone marrow suppression, including thrombocytopenia. In some cases, this thrombocytopenia can become immunological as the body begins to destroy its platelets [163,168]. Chemotherapy can also trigger an immunological response that contributes to the development of ITP. Patients treated with intensive chemotherapy are at increased risk of bleeding complications, which may require discontinuation of anticancer therapy or adjustment of drug doses [135]. Patients with ovarian cancer and concomitant ITP are at increased risk of bleeding, both external and internal, which can lead to serious complications, especially during surgical procedures, which are often part of ovarian cancer treatment. Bleeding can also delay chemotherapy treatment, which can negatively affect the effectiveness of cancer treatment. Furthermore, in patients with low platelet counts, the risk of bleeding in internal organs increases, which can lead to life-threatening complications [169,170].

Although cervical cancer rarely directly causes the development of ITP, immune dysfunction caused by the cancer or its treatment can trigger autoimmune processes leading to the destruction of platelets. Chemotherapy, especially with the use of platinum derivatives (e.g., cisplatin), often leads to thrombocytopenia, which in most cases is toxic. However, in some patients, autoimmune mechanisms of platelet destruction can develop, leading to the development of ITP. Additionally, with the development of immunotherapy, including ICIs such as pembrolizumab, the risk of autoimmune complications, including ITP, has increased. Immunotherapy, which enhances the body’s immune response, can trigger an excessive autoimmune reaction leading to the destruction of platelets. This in turn increases the risk of serious bleeding, which is particularly important for patients with cervical cancer who are already weakened by intensive anticancer therapy [161,171,172,173]. Similar mechanisms are observed in the treatment of endometrial cancer [148,174,175].

In a retrospective cohort study of 3,258,677 U.S. veterans, the incidence of ITP in patients with prostate cancer was 12.3/100,000 patient-years, whereas in patients without prostate cancer, it was 9.5/100,000 patient-years. Although the analysis showed a 30% higher risk of ITP in patients with prostate cancer, after adjusting for confounding factors such as age, this difference was not statistically significant. In a detailed review of data from the Memphis Veterans Administration Medical Center, using expanded ICD codes, six cases of ITP were identified in 3973 patients with prostate cancer. In contrast, no cases of ITP occurred in the control group (4801 patients without prostate cancer). The clinical course of ITP in patients with prostate cancer was similar to that seen in other autoimmune disorders and did not show a clear association with prostate cancer progression. Treatment for ITP included steroids, immunosuppressive drugs, and splenectomy in more severe cases. The results suggest that the association between ITP and prostate cancer may be due to confounding factors such as patient age. Still, the number of cases was too small to be clinically significant [176].

ITP in patients with metastatic cancer, especially breast and lung cancer, develops as a result of complex interactions between bone marrow dysfunction and autoimmune reactions caused by the tumor and its treatment [134,177,178,179]. Bone marrow metastases, which are common in advanced stages of these tumors, lead to disruptions in the bone marrow microenvironment, which negatively affect the production of blood cells, including platelets. As a result of bone marrow infiltration by tumor cells, there is direct damage to megakaryocytes, which are responsible for platelet production [180,181]. This, in turn, results in significant thrombocytopenia, which, in combination with autoimmune mechanisms, contributes to the development of ITP. Bone marrow metastases not only lead to impaired hematopoiesis but can also stimulate the body’s immune reactions. As a result, the immune system can begin to produce autoantibodies against platelets, accelerating their destruction. In such cases, patients develop an autoimmune form of thrombocytopenia, which is typical of ITP. In patients with breast and lung cancer, in whom bone marrow metastases lead to a reduced platelet count, the risk of serious complications, such as bleeding, becomes particularly significant [129,182,183].

Treatment of these cancers, including chemotherapy, contributes to the severity of thrombocytopenia. Cytostatic drugs such as paclitaxel or gemcitabine, which are often used in the treatment of breast and lung cancer, can cause myelosuppression and inhibit platelet production in the bone marrow. At the same time, they can induce autoimmune mechanisms that contribute to the destruction of circulating platelets. In these patients, the coexistence of chemotherapy-induced thrombocytopenia and autoimmune destruction of platelets can lead to a significant reduction in their number, resulting in the risk of serious hemorrhages [184,185,186].

Cases of ITP development in breast and lung cancer are particularly difficult to treat because they require simultaneous control of the neoplastic disease and autoimmune complications. Immunosuppressive therapy, used to suppress autoimmune responses, may impair the host response to the tumor, which requires a cautious and individualized approach to treatment. Monitoring platelet counts and promptly implementing appropriate interventions, such as corticosteroids or immunoglobulins, is important to prevent bleeding complications. In the context of breast and lung cancer with bone marrow metastases, ITP represents a significant clinical challenge that requires collaboration between oncologists, hematologists, and specialists in the treatment of autoimmune blood diseases to manage both the tumor and its hematologic complications optimally.

## 4. Surgical Interventions and ITP

**Figure 5:**
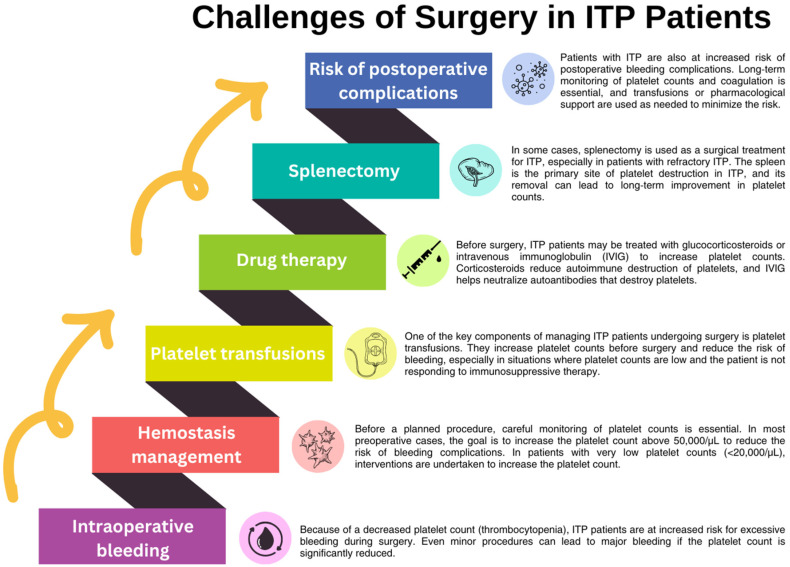
Challenges in surgical procedures in patients with ITP (based on [187,188,189,190]).

Surgical procedures in patients with ITP, especially in oncology patients, pose a serious clinical challenge due to the increased risk of bleeding complications (Figure 5). This is reflected in both trauma surgery procedures and oncology patients treated surgically. Optimizing the patient’s condition before the procedure by increasing the platelet count and controlling the immune response is crucial to minimizing the risk of bleeding. At the same time, careful monitoring of hemostasis in the perioperative and postoperative periods is essential. This is especially true for oncology patients, in whom additional therapies such as chemotherapy or immunotherapy can deepen thrombocytopenia and complicate the healing process. For this reason, appropriate management of the bleeding risk and individualization of treatment are essential to safely performing procedures in this group of patients [187,188,189,190].

Surgical procedures in cancer patients pose a unique challenge, resulting from the patient’s general health condition, coexisting diseases, and the effects of anticancer treatment. Cancer often leads to a weakened immune system, coagulation disorders, and reduced ability to regenerate tissue, which significantly increases the risk of surgical complications. Coagulation disorders, such as thrombocytopenia, are particularly dangerous in patients undergoing chemotherapy or radiotherapy, as they increase the risk of bleeding both during the procedure and in the postoperative period [191,192]. An additional problem is targeted therapies, such as angiogenesis inhibitors (e.g., bevacizumab), which can worsen clotting problems and slow the wound healing process [193,194,195].

A weakened immune system, resulting from both cancer and its treatment, significantly increases the risk of postoperative infections. These patients are more susceptible to infections, which is why careful monitoring, appropriate asepsis during surgery, and the use of antibiotic prophylaxis are necessary to reduce the risk of infections [196,197,198]. In addition, oncological treatment, especially long-term chemotherapy, negatively affects the body’s ability to regenerate tissue, which results in longer wound healing times and increases the risk of complications such as fistulas or anastomotic failure in gastrointestinal surgery. Many oncological patients undergo systemic treatment, affecting the healing process and the body’s overall metabolic state [199,200]. Immunotherapy, an increasingly popular method of treating cancer, can lead to autoimmune complications such as inflammation of various organs, which additionally complicates surgical procedures and the post-operative recovery period [201,202,203]. Therefore, surgical procedures in oncological patients require an individual approach, careful planning, and multidisciplinary care to minimize the risk of complications and provide optimal conditions for healing and recovery.

## 5. Treatment and Management of ITP in Oncology Patients

In cancer patients with ITP, a key element in managing the surgical risk of bleeding is appropriate preoperative preparation, which includes assessment of platelet counts and interventions to increase platelet counts. Treatment may include intravenous IVIG, corticosteroids, or thrombopoietin receptor agonists (e.g., eltrombopag) to increase platelet counts before surgery and minimize the risk of bleeding complications [204,205,206]. In the case of gynecologic, gastrointestinal, or other surgical procedures in cancer patients with ITP, close monitoring of the patient’s condition both preoperatively and postoperatively is essential to intervene promptly if necessary. Platelet transfusions (especially if platelet counts are very low (<30,000/µL)), as well as immunosuppressive therapy, are standard interventions in cases of profound thrombocytopenia [188,207].

The treatment of ITP in cancer patients requires particular caution, especially in interactions with anticancer therapies. Corticosteroids, such as prednisone and dexamethasone, are the standard first-line therapy for ITP. They work by reducing platelet destruction and modulating the immune response. Although corticosteroids are very effective in rapidly raising platelet counts, their long-term use is associated with serious side effects, including osteoporosis, diabetes, and increased risk of infection, which can be particularly challenging for cancer patients already weakened by cancer therapy [208,209,210].

In cases where corticosteroids are ineffective or sudden bleeding occurs, intravenous IVIG is used. IVIG works by blocking Fc receptors on macrophages, which inhibits platelet destruction. IVIG produces rapid but short-lived effects, meaning treatment must be repeated regularly. This approach is essential for cancer patients who need to quickly increase their platelet counts before surgery or cancer therapy [211,212,213].

Thrombopoietin receptor agonists (TPO-RAs), such as romiplostim and eltrombopag, are used to treat chronic ITP, especially in patients who do not respond to first-line therapy. These drugs mimic the effects of endogenous thrombopoietin, stimulating megakaryocytes to produce platelets. Studies suggest that TPO-RA is effective in maintaining platelet counts at safe levels in most patients, which is crucial for cancer patients who require long-term support for hematopoietic function [214,215,216]. Rituximab, a monoclonal antibody directed against the CD20 antigen, is often used off-label to treat ITP. It works by reducing the number of B cells responsible for producing antiplatelet autoantibodies. Although rituximab therapy is effective in 40–70% of patients, durable remission is rare, with only about 21% of patients maintaining a response to treatment after five years. In oncological patients, treatment with rituximab must be carefully monitored, especially due to the risk of complications related to the weakening of the immune system, which may additionally exacerbate the effects of immunosuppression associated with oncological therapies [75,217]. Examples of medications used to treat ITP are presented in Table 2.

Immunosuppressive and immunomodulating drugs, such as MMF (mycophenolate mofetil), cyclosporine A, azathioprine, danazol, dapsone, and hydroxychloroquine, are used to treat immune thrombocytopenic purpura (ITP), especially in patients who do not respond to standard therapies such as corticosteroids and intravenous IVIG. Table 3 provides a summary of their effects.

Studies suggest that IL-35, through its immunomodulatory properties, can inhibit the activity of Th1 and Th17 cells and reduce the production of proinflammatory cytokines such as IL-17, which play a crucial role in the pathogenesis of ITP. This makes IL-35 a potential therapeutic target for treating primary ITP, as inhibition of these signaling pathways can reduce autoimmune platelet destruction.

New drugs such as rilzabrutinib, an inhibitor of Bruton’s tyrosine kinase (BTK), have shown promising results in phase I/II clinical trials. Rilzabrutinib works by blocking the BTK pathway, which is crucial for activating B cells responsible for producing autoantibodies. In clinical trials, this drug achieved a response rate of 40% in patients with refractory ITP, suggesting its potential efficacy in difficult-to-treat cases [75].

Another innovative approach is using efgartigimod, an FcRn receptor inhibitor that lowers the level of IgG immunoglobulins. Since autoantibodies in ITP are mainly of the IgG type, blocking this receptor may reduce their number in circulation, which leads to the protection of platelets from immune destruction. Preliminary results indicate that efgartigimod may be effective in many autoimmune diseases, including ITP, making it a promising therapeutic option.

Sutimlimab, a complement inhibitor, aims to block the early steps of complement activation, which plays an important role in the pathogenesis of ITP. In phase I studies, sutimlimab achieved a response rate of 42% in patients with chronic, refractory ITP, indicating its potential as an innovative therapy in this difficult-to-treat group of patients [75].

Daratumumab, an anti-CD38 monoclonal antibody, is another new therapy that has shown efficacy in eliminating long-lived plasma cells responsible for producing antiplatelet antibodies. Daratumumab, primarily used in the treatment of multiple myeloma, is being studied in clinical trials as a potential therapy for ITP, where eliminating plasma cells can significantly reduce the production of harmful autoantibodies [75]. This could make a significant difference for patients with chronic and refractory ITP.

These new therapies offer hope for the future of ITP treatment, offering patients and physicians new tools to combat the disease. While traditional therapies such as corticosteroids and immunoglobulins may not be sufficient for refractory ITP, novel approaches, including TPO-RA and rituximab, offer patients new therapeutic options. Further research into the immunological mechanisms may lead to even more targeted therapies that are more effective and safer for patients. Thanks to advances in medicine, strict adherence to clinical guidelines and personalized therapeutic strategies can improve treatment outcomes and the quality of life of patients with ITP [75,218,219].

**Table 2:** Examples of drugs used in the treatment of ITP with particular emphasis on oncological patients.

Drug	Drug Category	Dosage	Effect	Target	Comments	References
Prednisone	Glucocorticosteroid	1–2 mg/kg body weight daily	It suppresses the immune response by inhibiting the production of cytokines like IL-2 and reducing macrophage activation.	T cells, cytokines, and macrophages	Monitoring is necessary due to the impact on the course of cancer. Moreover, long-term use is associated with the risk of complications such as osteoporosis, infections, and diabetes	[20,219,220]
Prednisolone	Glucocorticosteroid	1–2 mg/kg body weight daily. The initial dose may be maintained for 1–2 weeks and then gradually reduced depending on the patient’s clinical response.	It is a glucocorticosteroid that has anti-inflammatory and immunosuppressive effects, reducing the destruction of platelets by the immune system. It inhibits the production of autoantibodies and reduces the activation of macrophages, which are responsible for the phagocytosis of platelets.	Capillary endothelium.	Prednisolone is often used as a first-line treatment for ITP. In cancer patients, prednisolone can effectively quickly raise platelet counts, which is essential before surgery or during chemotherapy. However, its long-term use is associated with the risk of serious side effects, such as osteoporosis, hyperglycemia, hypertension, and increased susceptibility to infections.	[221]
Methylprednisolone	Glucocorticosteroid	0.5–1 mg/kg body weight per day, but in severe cases of ITP, a higher dose, even 1–2 mg/kg body weight per day, may be used. In pulse therapy (especially in acute cases), doses of 500 mg to 1 g for 3 days are used, especially in severe cases such as internal bleeding or refractory thrombocytopenia.	It has anti-inflammatory and immunosuppressive effects. Methylprednisolone inhibits the activation of T and B lymphocytes, reducing the production of autoantibodies and inhibits macrophages responsible for the destruction of platelets.	T cells, cytokines, leukocytes, and phospholipase A2	Methylprednisolone, mainly used in pulse form, is preferred in acute cases requiring rapid response. In situations of acute exacerbation of ITP, especially before surgery or in cases of bleeding, pulse doses of methylprednisolone can help to increase the platelet count quickly. In oncology patients, methylprednisolone may be an alternative to prednisolone, especially when high doses of corticosteroids are required. Pulse therapy with high doses of methylprednisolone may lead to fewer side effects than long-term low doses of prednisolone but requires intensive monitoring due to the risk of acute hyperglycemia, hypertension, and electrolyte disturbances.	[222]
Dexamethasone	Glucocorticosteroid	40 mg daily for 4 days (pulse dosing), repeated every 2–4 weeks	Increases the number of platelets by inhibiting their destruction through immune modulation.	Platelets and immune cells	Preferred short-term treatment for oncology patients	[223,224]
IVIG	Intravenous immunoglobulin	1 g/kg body weight daily for 1–2 days or 0.4 g/kg for 5 days; rapid but short-lasting effect	Binds to Fc receptors on macrophages, blocking their interaction with antibody-coated platelets, thus increasing circulating platelets	Fc receptors and macrophages	Used in emergencies. It has high costs and needs to be repeated.	[225]
Romiplostim	Peptide antibody	1 μg/kg body weight once a week; the dose may be increased to 10 μg/kg depending on the platelet count	It is a TPO-RA agonist that acts on megakaryocytes in the bone marrow to stimulate platelet production. It mimics the action of endogenous thrombopoietin, promoting megakaryocyte maturation and increasing platelet production.	Thrombopoietin receptor on megakaryocytes	Individualize the dose depending on the response, and pay attention to interactions with cancer therapy.	[208]
Eltrombopag	Thrombopoietin receptor agonists	50 mg once daily and 25 mg in patients with hepatic impairment or of Asian origin; avoid dietary calcium around administration	Binds to and activates the thrombopoietin receptor, promoting megakaryocyte differentiation and platelet production.	Thrombopoietin receptor on megakaryocytes	Regular monitoring of platelet counts, especially during chemotherapy.	[226]
Rituximab	Monoclonal anti-CD20 antibody	375 mg/m^2^ body surface area once weekly for 4 weeks	Targets CD20 protein on B cells, leading to their depletion and reducing autoantibody production against platelets.	B cells (CD20)	Requires monitoring due to risk of immunosuppression; effective in 40–70% of patients, but lasting remission is rare; risk of complications	[212]
Avatrombopag	Thrombopoietin receptor agonists	20 mg orally once daily. The dose may be increased depending on the patient’s response to treatment, with a maximum dose of 40 mg daily.	It is a TPO-RA agonist that stimulates platelet production by stimulating megakaryocytes. Its effects are similar to other TPO-RAs such as eltrombopag and romiplostim.	Bone marrow	Avatrombopag may be used in patients with ITP, especially those who require maintenance of adequate platelet levels during anticancer therapy. Avatrombopag has the advantage of not having significant interactions with food, which is an advantage over eltrombopag, which requires avoidance of dietary calcium.	[227]
Fostamatinib	Tyrosine kinase inhibitor	100 mg orally twice daily. If response to treatment is inadequate, the dose may be increased to 150 mg twice daily after one month of treatment.	It is a Syk kinase (spleen tyrosine kinase) inhibitor that inhibits intracellular signaling pathways responsible for platelet phagocytosis by macrophages. By inhibiting platelet destruction, fostamatinib enables their survival and maintenance of appropriate levels in the bloodstream.	SYK in macrophages	Particularly useful in patients with refractory ITP where other forms of therapy have been ineffective. Its unique mechanism of action differs from other drugs used in ITP, making it an alternative for patients who do not respond to corticosteroids-, IVIG-, or TPO-RA-based therapies.	[228]

**Table 3:** Other ITP therapeutic strategies.

Drug	Primary Indication	Dosage	Effect	Target	Comments	References
Mycophenolate mofetil (MMF)	It is primarily indicated for the prevention of organ transplant rejection by acting as an immunosuppressant drug that suppresses the activity of the immune system to protect the transplanted organ.	500 mg twice daily, which may be increased to 1 g twice daily depending on patient tolerance and response.	MMF acts as a purine synthesis inhibitor, which leads to a decrease in the proliferation of T and B lymphocytes, which play a key role in the pathogenesis of ITP. It inhibits the activity of immune cells responsible for the production of autoantibodies against platelets.	T cells, B cells	MMF is used as a second-line therapy in patients with chronic ITP, especially those who do not respond to other immunosuppressive drugs. MMF is well tolerated and has fewer side effects than some other immunosuppressive drugs. Regular monitoring of kidney function and white blood cell count is necessary, as the drug can lead to immunosuppression and an increased risk of infection.	[229]
Cyclosporine A	It is primarily indicated for the prevention of rejection of an organ transplant (e.g., kidney, heart, and liver). It acts as a powerful immunosuppressant drug that inhibits the activity of T lymphocytes, preventing the immune system from attacking the transplanted organ.	3–5 mg/kg body weight daily, adjusted depending on patient response and blood cyclosporine levels.	It is a calcineurin inhibitor, which inhibits the activation and proliferation of T lymphocytes, reducing their ability to stimulate an immune response. In ITP, this drug reduces the production of autoantibodies against platelets.	T cells, cytokines	It requires monitoring of the drug concentration in the blood, as it can lead to nephrotoxicity and other side effects, such as hypertension and hypertrichosis. The use of this drug is also associated with the risk of immunosuppression.	[230]
Azathioprine	It is primarily indicated for the prevention of organ transplant rejection and the treatment of autoimmune diseases such as rheumatoid arthritis, systemic lupus erythematosus, and Crohn’s disease. It acts as an immunosuppressant, inhibiting the activity of the immune system by blocking the proliferation of lymphocytes.	1–2 mg/kg body weight per day. The dose may be adjusted depending on the tolerability and effectiveness of the treatment.	It is an immunosuppressive drug that works by inhibiting DNA synthesis in lymphocytes, which reduces their proliferation and activity. It limits the production of autoantibodies against platelets, which helps in the treatment of ITP.	T cells, B cells	Azathioprine therapy requires a long time to achieve a clinical effect. Blood counts must be monitored because azathioprine can cause myelosuppression. Regular liver function tests are also important because the drug can be hepatotoxic.	[231]
Danazol	It is used to treat endometriosis, hereditary angioedema, and ITP. It works by inhibiting hormone production and supporting the immune system, reducing the symptoms of these conditions.	200–400 mg daily, depending on patient response. The dose may be gradually reduced once an adequate platelet count has been achieved.	Synthetic androgen acts immunomodulatory by reducing the production of autoantibodies and improving platelet count. It inhibits the production of IL-1 and TNF by monocytes, modulating the immune response.	IL-1, TNF, monocytes	It may lead to side effects related to sex hormones, such as virilization in women, voice changes, as well as hepatotoxicity, so liver function should be monitored.	[232]
Dapsone	It is primarily indicated for the treatment of leprosy (Hansen’s disease) and *Pneumocystis jirovecii* pneumonia in immunocompromised patients, often as an alternative to trimethoprim-sulfamethoxazole. It acts as an antibacterial and anti-inflammatory drug. Additionally, it is used in some autoimmune diseases, such as pemphigus and dermatitis herpetiformis, due to its anti-inflammatory and immunosuppressive properties	50–100 mg daily.	It is an anti-inflammatory drug that has immunomodulatory effects, although its exact mechanism in ITP is not fully understood. It is known to reduce the production of autoantibodies and improve platelet count. Induces hemolysis, leading to erythrophagocytosis in the reticuloendothelial system and thus preventing platelet destruction.	Reticuloendothelial system	The drug may cause side effects such as hemolytic anemia and methemoglobinemia, so regular monitoring of blood counts is necessary.	[233]
Hydroxychloroquine	It is primarily indicated for the treatment of malaria and autoimmune diseases such as SLE and rheumatoid arthritis (RA). It acts as an anti-inflammatory and immunomodulatory drug, inhibiting the activity of the immune system and reducing the symptoms of inflammation in these diseases.	200–400 mg daily, depending on the patient’s condition.	It is an immunomodulatory drug that inhibits the activity of immune cells, reducing the production of autoantibodies. It is mainly used in autoimmune diseases such as lupus erythematosus, but can be used in ITP to reduce the destruction of platelets.	Platelets, vascular system	Regular monitoring of eyesight is necessary, as long-term use of hydroxychloroquine may lead to retinopathy.	[234]

Treatment of ITP in cancer patients requires special caution due to the already weakened immune system, the risk of complications resulting from anticancer therapy, and the potential for side effects to be exacerbated by drugs used in ITP. Cancer patients are often more susceptible to infections, myelosuppression, organ failure, and other complications, making many drugs used in ITP contraindicated or requiring close monitoring. In this regard, the following is a discussion of the side effects and contraindications of key drugs used in ITP therapy in cancer patients. The first example is prednisone, commonly used in the treatment of ITP, which increases the risk of infections, especially fungal and viral infections, which is a serious threat to patients with weakened immune systems. The use of prednisone in cancer patients can lead to serious, potentially life-threatening infections. In addition, prednisone can cause hyperglycemia, which is particularly important in cancer patients, who often suffer from metabolic disorders, including diabetes induced by anticancer therapy such as steroids or mTOR inhibitors. Long-term use of prednisone can increase insulin resistance and lead to uncontrolled hyperglycemia. Corticosteroids can also contribute to bone loss and osteoporosis. Cancer patients, especially those who have undergone chemotherapy or radiotherapy, are particularly susceptible to bone weakness, which increases the risk of fractures. Moreover, prednisone can cause an increase in blood pressure, which is particularly dangerous in patients with hypertension caused by other anticancer drugs, such as VEGF inhibitors [235,236,237]. Another drug is MMF, which can cause severe myelosuppression, which is particularly problematic in cancer patients who may already have low blood counts due to chemotherapy. Low levels of white blood cells and lymphocytes increase the risk of infections, including severe bacterial, viral, and fungal infections. In cancer patients with active infections, the use of MMF can worsen the course of the disease, increasing the risk of sepsis and death. In addition, MMF can lead to ulceration and bleeding in the gastrointestinal tract, which is risky, especially in patients with gastrointestinal tumors or damage to the intestinal mucosa caused by chemotherapy [238,239,240]. Cyclosporine A, due to its nephrotoxicity, can lead to kidney damage, which is particularly important in cancer patients who may already have impaired kidney function due to therapy with nephrotoxic drugs such as cisplatin. Monitoring kidney function is therefore crucial in these patients. In addition, cyclosporine strongly inhibits T-cell responses, which significantly increases the risk of infections. Cancer patients, especially those undergoing chemotherapy or with weakened immune systems, are particularly susceptible to developing serious infections, including fungal, viral (e.g., EBV, CMV reactivation), and bacterial infections. Additionally, cyclosporine can cause an increase in blood pressure, which can be problematic in cancer patients with hypertension induced by other drugs [241,242,243]. Another drug is azathioprine, the use of which can cause severe myelosuppression, which is a serious risk for cancer patients who are already exposed to myelosuppression associated with chemotherapy. This increases the risk of infections, anemia, and thrombocytopenia. Long-term use of azathioprine is also associated with an increased risk of developing malignancies, such as skin cancer and lymphomas. For cancer patients who are already at risk of developing secondary malignancies due to anticancer therapy, this risk is particularly significant. In addition, azathioprine can lead to hepatotoxicity, which is particularly problematic in patients with liver damage caused by tumors, metastases, or hepatotoxic drugs [244,245]. Studies indicate that danazol can cause serious liver damage, including cholestatic jaundice and liver failure, making its use risky in patients with liver tumors or liver metastases. Danazol also increases the risk of thrombotic complications, which is particularly dangerous in cancer patients who are already at high risk for thrombosis due to anticancer therapy such as VEGF inhibitors and tumor-induced inflammation. In addition, in cancer patients, especially women, danazol can lead to adverse androgenic effects such as hirsutism, acne, and voice changes [246,247]. Another example is dapsone, the administration of which can cause hemolysis in patients with glucose-6-phosphate dehydrogenase deficiency, which is of particular concern in cancer patients with anemia, in whom further hemolysis could worsen the clinical condition. Dapsone can also lead to methemoglobinemia, which can be dangerous in patients with lung cancer or in those whose oxygen-carrying capacity is already limited by other cancers [248,249]. A final example is rituximab, which can cause reactivation of viruses such as HBV and CMV, which can lead to severe infections in patients with weakened immune systems. In addition, rituximab inhibits the production of blood cells in the bone marrow, which increases the risk of infections, anemia, and severe thrombocytopenia, especially in patients undergoing chemotherapy [250,251,252].

## 6. Conclusions

ITP is a complex autoimmune disease in which the immune system destroys platelets, leading to thrombocytopenia and an increased risk of bleeding. The pathogenesis of ITP involves dysregulation of various components of the immune system, including T and B lymphocytes, macrophages, and cytokines, which lead to the production of autoantibodies against platelets. Autoreactive T cells and an imbalance between Th1, Th17, and Treg lymphocytes play a key role in the exacerbation of the autoimmune process, and aberrant signaling of cytokines such as IL-2, IL-4, IL-10, and TNF-α additionally contribute to platelet destruction. In the context of oncology patients, ITP can be both primary and secondary. Secondary ITP often develops as a result of anticancer therapies such as chemotherapy or immunotherapy, which can trigger autoimmune reactions. The treatment of cancer patients with ITP requires an individualized approach, taking into account the interactions between anticancer therapies and drugs used for ITP. Corticosteroids, TPO-RA, and new therapies such as rituximab can be used effectively, although they require close monitoring to minimize the risk of complications such as thrombocytosis, infections, or hypertension. Surgical interventions in patients with ITP pose an additional challenge, as thrombocytopenia increases the risk of bleeding complications both during and after surgery. Assessment of the risk of bleeding, monitoring of platelet counts, and the use of platelet-enhancing therapies such as IVIG or TPO-RA are key to improving surgical outcomes. Particularly in cancer patients, where anticancer therapies can further weaken the immune system, a multidisciplinary approach is necessary to minimize the risk of complications. In summary, the treatment of ITP requires a flexible approach that takes into account the severity of the disease, the response to previous therapies, and comorbidities such as malignancies. Modern immunosuppressive and immunomodulatory therapies offer hope for improving outcomes in patients with chronic and refractory ITP. Further research into immunological mechanisms may yield new, more effective treatments that will be better tailored to individual patient needs.

## Figures and Tables

**Table 1:** Comparison of primary and secondary ITP (based on [14,17,24,25,26,27,28,29,30,31,32,33,34,35,36,37]).

	Primary ITP	Secondary ITP
Definition	This is a form of ITP in which no clear cause can be identified. It is a diagnosis of exclusion, meaning that other causes of thrombocytopenia must be ruled out before it can be diagnosed.	In this case, thrombocytopenia develops as a result of known predisposing factors, such as other diseases, infections, medications, or tumors. Secondary ITP is a reaction to these factors and is not a primary autonomic disorder.
Etiology	Pathogenesis is associated with an abnormal immune response against one’s own platelets. This leads to the formation of autoantibodies against platelet surface antigens, leading to their destruction, mainly in the spleen. The exact cause of this immune response is unknown, making this type idiopathic.	It has well-defined causes, such as follows: Autoimmune diseases: systemic lupus erythematosus (SLE) and antiphospholipid syndrome. Infections: viral infections, such as HIV and HCV. Medications: some medications can induce secondary ITP, for example, heparin (causing HIT—heparin-induced thrombocytopenia). Cancers: Some cancers, especially lymphomas and other hematological malignancies, can lead to secondary ITP. Pregnancy: pregnancy status can also predispose to the development of secondary ITP.
Immunological mechanism	Autoantibodies, mainly IgG, bind to platelet surface antigens such as glycoproteins GPIIb/IIIa and GPIb/IX. Coating platelets with these antibodies leads to their recognition by Fcγ receptors on the surface of macrophages, which facilitates their phagocytosis, mainly in the spleen and liver. This mechanism shortens the lifespan of platelets from the normal 7–10 days to only a few hours, leading to a significant reduction in platelet counts in peripheral blood and, consequently, to thrombocytopenia. In addition to destroying circulating platelets, ITP can also affect megakaryocytes, the precursor cells responsible for platelet production in the bone marrow. Autoantibodies can bind to megakaryocytes, inhibiting their maturation and production of new platelets. As a result, ITP is associated with excessive platelet destruction and reduced production of new platelets, which further worsens thrombocytopenia	The immunological mechanism is similar but results from immune activation as a result of another disease. These include the destruction of megakaryocyte precursors by the immune system, exposure to certain drugs, toxins, infections, and other disease states such as aplastic anemia. The resulting reduction in megakaryocyte precursors and ineffective regulation of thrombopoiesis may lead to secondary ITP. Factors that may reduce the number of megakaryocyte precursors include cancer, such as myelodysplasia, acute leukemia, or metastatic bone marrow disease, which can disrupt the average production and function of these cells. In these cases, the bone marrow environment is altered, leading to impaired megakaryopoiesis. In addition, the destruction of megakaryocyte precursors by the immune system, exposure to certain toxins such as benzene, and the use of certain drugs such as quinine can further reduce their numbers.
Clinical course	It can be acute or chronic. In children, it is often acute and transient, and in adults, it is more often chronic. In patients with chronic primary ITP, treatment can be long-term, and the disease is characterized by recurrent episodes of thrombocytopenia.	It usually has a more chronic course than primary ITP. Prognosis depends on the underlying disease, and ITP often resolves with successful treatment of the secondary cause (e.g., eradication of infection, control of autoimmune disease, and discontinuation of medication).
